# Do lifestyle and hormonal variables explain links between health and facial attractiveness?

**DOI:** 10.3389/fpsyg.2024.1404387

**Published:** 2024-08-14

**Authors:** Steven Arnocky, Adam C. Davis

**Affiliations:** ^1^Human Evolution Laboratory, Department of Psychology, Nipissing University, North Bay, ON, Canada; ^2^Department of Social Sciences, Canadore College, North Bay, ON, Canada

**Keywords:** facial attractiveness, immunocompetence, good genes sexual selection, unhealthy lifestyle, skinfold, secondary sexual characteristics

## Abstract

**Introduction:**

Facial attractiveness has recently been considered an indicator of underlying immunocompetence. However, studies examining this relationship have yielded mixed findings. Previous research suggested that these discrepant findings could be due to the common influence of lifestyle factors upon both rated facial attractiveness and health.

**Methods:**

Young men (*N* = 162) provided standardized facial photos with a neutral expression subsequently rated by eight women for overall attractiveness. Saliva was assayed for immunoglobulin A, testosterone (T) and cortisol (C), and body fat was measured using a skinfold caliper. Self-reports of poor health, and lifestyle factors that could influence health status (age, sleep habits, smoking, drinking alcohol, family stress, and exercising) were collected.

**Results:**

Results showed that symptoms of poor health and skinfold negatively predicted facial attractiveness. There was a modest but statistically non-significant T x C interaction where higher T lower C men trended toward having more attractive faces. A sequential mediation model examining the influence of lifestyle showed support for an indirect effect on facial attractiveness. Specifically, skinfold and poor health symptoms mediated the links between exercise, stress, and facial attractiveness.

**Discussion:**

These findings suggest links between facial attractiveness and immunocompetence could be linked to some common lifestyle and hormonal variables, but that more comprehensive research involving lifestyle indicators (such as nutrition) are necessary.

## 1 Introduction

Humans are remarkably consistent in their assessment of what constitutes an attractive face (Langlois et al., 1991, 2000; Cunningham et al., 1995). Symmetry, averageness, skin quality, and hormone-linked sexually dimorphic features together form a facial structure that may be considered along a spectrum of attractiveness to the opposite sex (Arnocky et al., 2014). Faces may be an important source of reproductively relevant information accessible within a small amount of space (see Arnocky et al., 2014 for review). Specifically, attractive faces are believed to serve as a cue to an individual’s genotypic quality (e.g., Hume and Montgomerie, 2001). Indeed, some previous research has reported positive links between facial attractiveness and health (see Arnocky et al., 2014; Jones et al., 2021 for review). From this perspective, ancestors who happened to prefer immunocompetence-linked morphological traits, such as those contributing to facial attractiveness, would have mated with partners who were better able to survive, accrue resources, and successfully rear offspring, and to have produced healthier offspring who themselves would be more likely to survive and reproduce.

In support of the facial attractiveness immunocompetence hypothesis, Shackelford and Larsen (1997) found that facial asymmetry was linked with poorer health among men and women. Hume and Montgomerie (2001) found that female facial attractiveness was tied to their body mass index (BMI) and health history, whereas facial attractiveness in men was linked to their childhood socioeconomic standing, which could indicate a role of environmental or lifestyle factors affecting facial development. There is also circumstantial evidence suggesting a putative link between facial attractiveness and immunocompetence. Humans tend to reliably rate more attractive faces as being healthier. For instance, Fink et al. (2006) found that female faces that were more symmetrical were also more attractive, but also were perceived as healthier. Foo et al. (2020) found that facial traits contributing to overall attractiveness, such as averageness, symmetry, skin yellowness, and adiposity in men, predicted raters’ perceptions of the health of those faces. Facial attractiveness is also tied to mating success in some studies: men with attractive faces have more short-term sex partners, and women with attractive faces start having sex at an earlier age and have more long-term sex partners (Rhodes et al., 2005). Some research suggests that there may be a sex difference in the link between facial attractiveness and immunocompetence. For example, men (Rantala et al., 2012), but not women (Rantala et al., 2013), with attractive faces have a stronger immune response to a hepatitis vaccine.

Still, other research has found null links between facial attractiveness and health. Kalick et al. (1998) examined the relationship between adult health and their rated facial attractiveness at late adolescence. They found no links across the lifespan. Nevertheless, raters inaccurately perceived attractive faces as being healthier within the sample. Similar findings were observed by Foo et al. (2020), where (as described earlier), attractive faces were viewed as healthier by raters, yet facial attractiveness was nevertheless unrelated to markers of immunocompetence including oxidative stress and lysozyme activity. Other research using a large (> 4,000 participant) sample found no links between longitudinal measures of childhood health and facial asymmetry (Pound et al., 2014). More recently, Cai et al. (2019) found that neither female facial attractiveness, sexual dimorphism, averageness, or coloration predicted self-reported health or salivary immunoglobulin-A (sIgA). Similarly, other work found that male facial attractiveness did not predict antibody levels following vaccination (Pátková et al., 2022).

### 1.1 Considering potentially important covariates

Jones et al. (2021) recently suggested that the discordant findings pertaining to the link between facial attractiveness and health might be due to covariates that could impact both variables. Specifically, they proposed that “rather than reflecting immunocompetence, facial attractiveness is instead more closely linked to aspects of lifestyle that produce health benefits” (pp. 3). The researchers argued that lifestyle factors, which can vary intra-individually over time, might explain changes in individuals’ facial attractiveness over time. Which lifestyle factors are relevant to facial attractiveness and health? Jones et al. (2021) focused on the examples of diet and body fat, which certainly have implications for health status and may have a stronger link to facial attractiveness than do markers of immunocompetence (Cai et al., 2019).

Rantala et al. (2013) found that body fat was curvilinearly related to facial attractiveness: Women with low or high body fat were rated as less attractive than those having intermediate body fat. Exercise also has well-established links to health (e.g., Akimoto et al., 2003; Murphy et al., 2009). Diets rich in highly processed and refined foods, typical of Western populations, have been linked to a range of physical and mental health problems (Cordain et al., 2005). Focusing on unhealthy dietary habits, Visine et al. (2024) found that consumption of food high in refined carbohydrates with a high glycemic load was associated with reduced facial attractiveness (rated by opposite-sex others) in both women and men. These effects remained after controlling for potential confounds, including age, sexual dimorphism, BMI, physical activity, smoking, and relationship status. Despite well-established links between exercise and health (e.g., Murphy et al., 2009), the link between exercise and facial attractiveness is less clear. Hönekopp et al. (2010) found that a composite measure of physical fitness predicted rated body but not facial attractiveness. Yet other research has shown that higher performance athletes are rated as being more facially attractive (e.g., Bagozzi et al., 2018). Moreover, men with stronger grip strength are rated as being more facially attractive (Fink et al., 2007).

Other candidates include exposure to smoke and alcohol, which when used in excess are known to have widespread negative health consequences (see Hurley et al., 2012 for review). Prototype faces of identical twins who smoke are rated less attractive than the non-smoking twin images (Skinner et al., 2017). Likely mechanisms of smoking-related change in attractiveness include skin wrinkling, pale-yellow (i.e., sallow) complexion, and gaunt facial structure (reviewed in Doshi et al., 2007). Some studies show that acute alcohol use can increase others’ ratings of the drinker’s facial attractiveness (e.g., Van Den Abbeele et al., 2015). Nevertheless, excessive alcohol use can lead to psoriasis, eczema, and skin infections (Higgins and Du Vivier, 1992) as well as jaundice, hyperpigmentation, and vascular issues including spider telangiectasias and angiomas (Liu et al., 2010).

Stress has also been implicated in both features influencing facial attractiveness (such as skin quality; see Koizumi et al., 2023 for review) and a diverse range of negative health consequences (see Apanius, 1998). Finally, sleep might also affect both facial attractiveness and health. Individuals photographed following 2 days of sleep restriction were rated as less attractive than when they had appropriate sleep. The researchers reasoned that aversion to mating with a sleep disturbed partner could help avoid sleep-related health issues (Sundelin et al., 2017).

Besides the study by Visine et al. (2024) described above, one other study to consider lifestyle factors in relation to facial attractiveness and health was conducted by Mengelkoch et al. (2022). These researchers examined rated facial attractiveness and various markers of health along with covariates, including BMI, adult socioeconomic status (SES), exercise, smoking behavior, and recent stress. However, given that these variables were not the primary focal point of the study, only those that were significantly related to rated attractiveness (BMI and age) were retained in their models. Their findings suggested that facial attractiveness was related with higher rates of phagocytosis and lower rates of bacterial growth in plasma, along with lower neutrophil counts, together suggesting better anti-bacterial immunity, but not with cellular proliferation or cytokine production.

### 1.2 Hormones

Hormones play an important role in coordinating phenotypic development (Roney, 2016) and therefore might also serve as important covariates when examining links between facial attractiveness and health. In a sample of young Latvian women, Rantala et al. (2013) found that facial attractiveness (as rated by men) was unrelated to the production of anti-hepatitis B surface antigen following a hepatitis B vaccination. However, they did find that (in addition to the body fat finding described earlier), women with high cortisol (C) had faces that were rated as less attractive. The authors considered that perhaps facial attractiveness serves as a cue to one’s exposure to, or ability to cope with, life stressors, or that low C also signals health in humans. Other research has found either null links between women’s facial attractiveness and C (Gonzalez-Santoyo et al., 2015, Study 1) or mixed results whereby some samples rate women with low C as having either more attractive (US raters) or less attractive (Mexican raters) faces (Gonzalez-Santoyo et al., 2015). Meta-analysis shows that flatter diurnal Cortisol slopes are associated with diverse negative health markers (Adam et al., 2017; see also: Knack et al., 2013). Nevertheless, comparatively less work has considered the role between men’s cortisol and their facial attractiveness.

Testosterone (T) is another hormone that may be complicit in both men’s health and facial attractiveness. Some research has shown that men with higher T are rated by women as having more attractive faces (e.g., Roney et al., 2006; Rantala et al., 2012), and T also has implications for immune functioning. For example, T is positively associated with sIgA in men (Arnocky et al., 2018; Hodges-Simeon et al., 2020). Yet other studies have found null links between T and rated male facial attractiveness (e.g., Swaddle and Reierson, 2002; Neave et al., 2003; Penton-Voak and Chen, 2004), and others have found null links between T, C, and both facial attractiveness and other-rated perceptions of health (Kandrik et al., 2017). Some researchers have suggested that relying on baseline T or C levels may be insufficient, and that the dual hormone hypothesis involving an interaction between high T and low C might be complicit in phenotypic masculinization. Indeed, Rantala et al. (2012) found that high T low C men’s facial photos were rated as most attractive by women. However, other research has failed to observe these effects (Kordsmeyer et al., 2019). For example, T, C, and percentage of adipose tissue were unrelated to ratings of men’s facial attractiveness (Pátková et al., 2022). Thus, more research is needed regarding the potential impacts of immunocompetence by hormone interactions on the development of phenotypic characteristics and the perceived attractiveness of those traits (Davis and Arnocky, 2022).

## 2 The present study

The goal of this research was to examine whether individual lifestyle factors, as well as abdominal skinfold measurements, that are theoretically common to both facial attractiveness and health might eliminate these links when controlled for in a regression analysis. In so doing, this research also aimed to examine whether previously reported links between facial attractiveness and biological and self-report markers of health are broadly replicable, given previously inconsistent findings.

Two indices of health were examined in the current study: Self-reports of poor health symptom frequency and severity and, following Cai et al. (2019), salivary immunoglobulin-A (sIgA). sIgA is a potentially important marker of underlying immunocompetence that is produced by plasma B cells, comprising over 70% of our mucosal antibodies (Macpherson et al., 2008) that provide an initial defense against pathogens (Macpherson et al., 2008; Brandtzaeg, 2009). Low levels of sIgA have been linked to increased infection (Fahlman and Engels, 2005; Nakamura et al., 2006; Volkmann and Weekes, 2006) as well as to self-reported severity and frequency of poor health symptoms in otherwise healthy young adult university students (Arnocky et al., 2023) and, longitudinally, to death in older adults (Phillips et al., 2015). sIgA has previously been linked to other apparently sexually selected phenotypic traits that may serve as cues to underlying immunocompetence, including the deep male voice (Arnocky et al., 2018) and female breast morphology (Locke and Arnocky, 2021).

**Figure 1:**
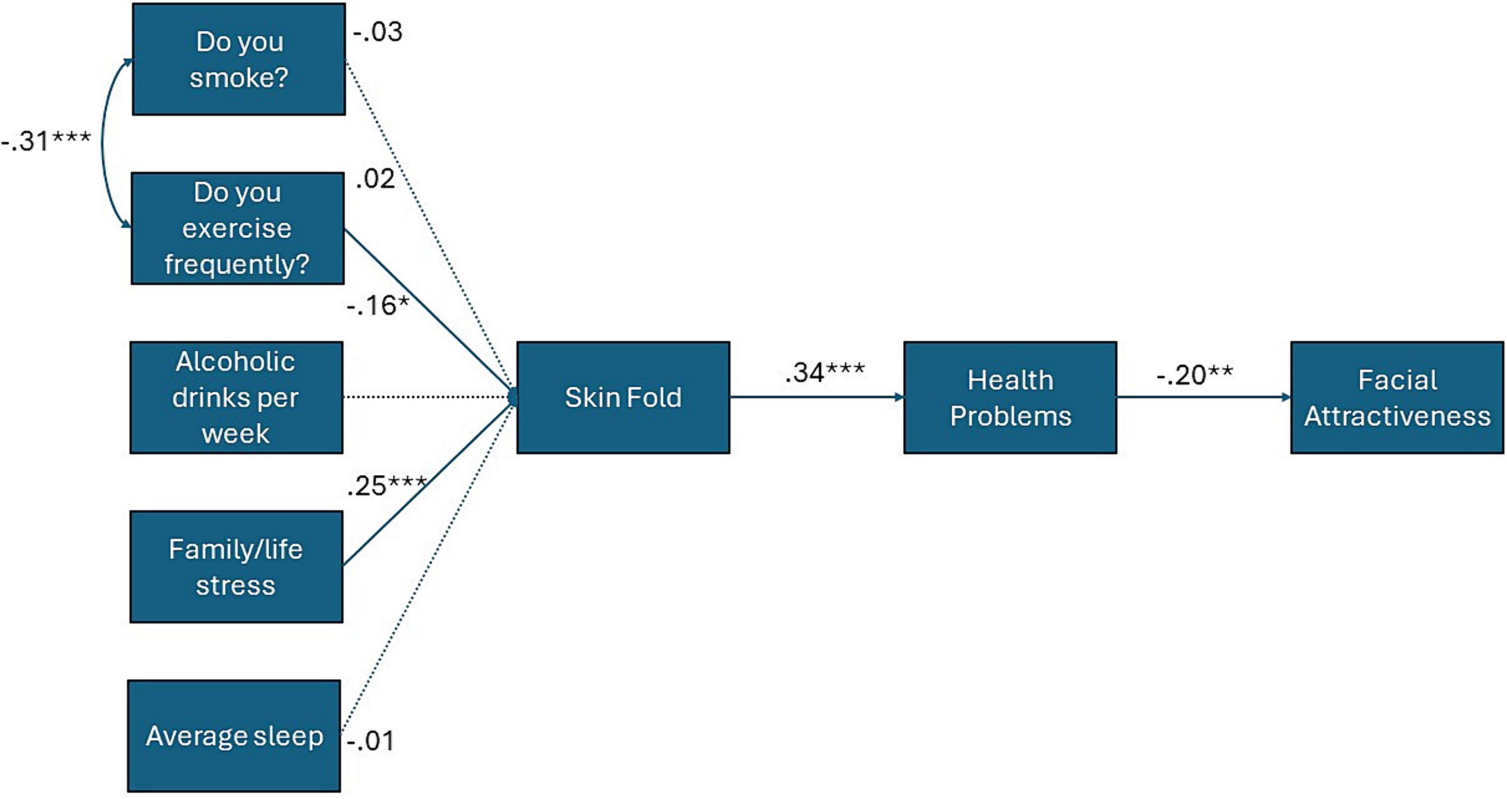
Path model for indirect effects of lifestyle variables upon facial attractiveness via skinfold and symptoms of poor health. Solid lines depict statistically significant paths, dashed lines depict statistically non-significant paths. Standardized coefficients shown. * = *p* < .05; ** = *p* < 0.01; *** = *p* < 0.001.

We expected an initial negative bivariate correlation between facial attractiveness and self-reported symptoms of poor health (Hypothesis 1A), and a positive correlation between facial attractiveness and sIgA (Hypothesis 1B). We then examined whether controlling for age (in years), lifestyle variables (sleep, familial stress, alcohol and tobacco use, exercise) and skinfold, along with hormones that have been linked to both health and facial attractiveness (T, C, and a T x C interaction), would weaken any observed links between symptoms of poor health, sIgA, and facial attractiveness (Hypothesis 2). Finally, we considered a sequential mediation model whereby unhealthy lifestyle habits have an indirect effect on facial attractiveness. Specifically, we expected that unhealthy lifestyle variables would predict a thicker abdominal skinfold, which has been identified as a predictor of future health problems in previous research (Loh et al., 2018). Therefore, in our model, skinfold was entered as a predictor of poor health symptoms, which in turn would predict lower rated facial attractiveness (Hypothesis 3, see Figure 1).

## 3 Materials and methods

### 3.1 Participants and procedure

As a part of a larger study on immune function and phenotypic development, males from a small Canadian University and the community were recruited through the institutional research participation system and posters in local businesses around town. A sample size calculation was performed using G*Power (3.1.9.7) with an expected medium effect size (*F^2^* = 0.15), 80% power, α = 0.05, and 12 predictors, which yielded a sample size of 127. The total sample in the existing data set was 162 young adult men, aged 18–39 years (*M_age_* = 22.7, SD = 4.7; 91.4% were students). The ethnic composition of the sample was Caucasian (90%), Black (4%), East Asian (3%), South Asian (2%), and Indigenous/First peoples (1%). Participants received either $50 CAD remuneration or partial course credit and $10. This research received approval by the Nipissing University Research Ethics Board (protocol # 100770–26,667).

### 3.2 Measures

#### 3.2.1 Hormones and immunoglobulin-a

Participants were asked not to eat, drink (except water), brush their teeth, or exercise 2 hrs prior to their testing session, and were rescheduled if they reported any current or recent acute symptoms of illness during their telephone screener prior to their session. Saliva samples were collected in 5 mL polystyrene culture tubes and stored at −80°C until assayed in duplicate via enzyme immunoassay kits (DRG International, NJ, United States) in the Principal Investigator’s lab. Sample provision time ranged between 8:30 AM and 5:00 PM. For sIgA, intra- and inter-assay CVs were below 6%. For T (pg/mL), intra-assay CV was below 4% and the inter-assay CV was below 8%. For C (ng/mL), the intra-assay CV was below 2% and the inter-assay CV was below 11%. To account for the typical non-normal distribution of these markers, the average of the duplicates was log-transformed. Given that salivary flow rate affects sIgA levels, we corrected the concentration value to reflect a flow (mL/s)-corrected μg/mL score (log-transformed). Sample provision time was related to C, and participant age was related to both C (*r* = −0.21, *p* = 0.007) and T (*r* = −0.24, *p* = 0.002), whereas sIgA was unrelated to either age or sample provision time.

#### 3.2.2 Unhealthy lifestyle

As part of a self-reported health screener, participants then completed items which addressed unhealthy lifestyle factors, including alcohol consumption (“*How many alcoholic beverages do you drink [on average] per week?*”), smoking/tobacco exposure (“*Do you smoke?”* [binary]), life stress (“*Have you or your family recently experienced any life changes or unusual psychological stress?*” [binary]), exercise (“*Do you exercise regularly?*” [binary]), along with sleep (“*How many hours do you sleep on average at night?*” [continuous]). A second indicator of unhealthy lifestyle was individuals’ skinfold measurement obtained using a digital body fat caliper. Thicker skinfold is associated with unhealthy eating habits from an early age (e.g., Dalrymple et al., 2019), including being linked to consumption of ultra-processed foods (Rohatgi et al., 2017), and is associated with a host of cardiometabolic risks in adulthood (González-Torres et al., 2023). Accordingly, skinfold has been used by researchers as an indicator of nutritional status (e.g., Bernstein et al., 2002). Body fat is highly correlated with facial adiposity (see Sierra-Johnson and Johnson, 2004) and plays an important role in determining male facial attractiveness (Windhager et al., 2011). The participant’s suprailliac skinfold (approximately one inch about the right hipbone) was measured three times and then averaged, (*α* = 0.99, 95% LLCI = 0.991, 95% ULCI = 0.995).

#### 3.2.3 Self-reported health

Self-reported health was assessed using The Health Symptoms Survey (Knack et al., 2011, 2012), which records both the frequency and severity of physical health problems. The measure demonstrates good construct validity, correlating with health-linked personality factors and behavioral issues (Knack et al., 2012), altered hypothalamic–pituitary–adrenal axis functioning (Knack et al., 2011), and sIgA as a biological marker of immunocompetence (Arnocky et al., 2023). The measure includes 56 items ranging from 1 (*Not at All*/*Does not Hurt at All*) to 4 (*All the Time*/*Unbearable Pain*) to determine the frequency and severity (28 items each) of symptoms, including stomach aches, flu, mouth sores, fatigue, chest pain, diarrhea, muscle aches and pains, headache or migraine, coughing, and fever experienced over the past year. A mean score was created with the measure demonstrating good internal consistency (*α* = 0.91, 95% LLCI = 0.77, 95% ULCI = 0.94).

#### 3.2.4 Facial attractiveness

Each male participant provided a standardized color photograph with a neutral facial expression. Photos were taken from a stationary camera (Canon EOS Rebel T6) in a well-lit room with no windows. Photos were in color and were 4,608 pixels wide by 3,456 pixels high. The facial stimuli took up most of the photo area, with only a small portion of the neck and shoulders visible. Photos were not edited in any manner, with the intention of having the ratings being made on naturalistic stimuli. These photos were rated by eight Caucasian women (*M*_age_ = 21, SD = 1.70) who were asked to report the level of facial attractiveness of each photo, presented in random order, using a Likert-type scale ranging from 1 = *Very unattractive*, to 10 = *Very attractive*. The raters were reliably consistent in their ratings for facial attractiveness (α = 0.82, 95% LLCI = 0.77, 95% ULCI = 0.86). Previous studies have demonstrated that researchers can obtain reliable attractiveness ratings using a small number of raters (e.g., Buss and Shackelford, 2008; Kordsmeyer et al., 2019).

## 4 Results

Descriptive statistics are presented in Table 1. Analyses were preformed using SPSS (29.0.1.0; IBM Corp, 2023). First, a bivariate correlation analysis (Table 2) was conducted to determine whether facial attractiveness correlated with the control variables (age, lifestyle factors, skinfold, and hormones) and the two health indicators (symptoms of poor health, sIgA). Age was negatively correlated with T, C, and their interaction, but was otherwise unrelated to lifestyle, health, and facial attractiveness. T and C were positively correlated. C was correlated negatively with exercise and positively with smoking, whereas T was unrelated to all lifestyle variables. Results showed that those with more attractive faces had lower abdominal skin fold values, fewer health problems, exercised more, and were modestly higher in sIgA.

**Table 1:** Descriptive statistics for study variable.

		*N*	*M*	SD	Min	Max
1. Age		160	22.71	4.71	18.00	39.00
2. Facial Attractiveness		162	4.00	1.28	1.25	7.75
3. Health Problems		162	1.35	0.25	1.00	2.29
4. sIgA		161	111.33	84.74	20.80	702.20
5. Skin Fold (mm)		162	14.26	8.44	3.30	41.23
6. Sleep		162	7.31	0.96	3.50	9.50
7. Exercise	Yes	133				
	No	29				
8. Alcohol (drink/week)		162	4.96	5.19	0.00	24.00
9. Smoking	Yes	9				
	No	153				
10. Life Stress	Yes	33				
	No	129				
11. Testosterone (T)		162	132.60	101.29	11.28	1155.50
12. Cortisol (C)		162	6.09	4.86	0.07	20.36

Biomarker (sIgA, T, C) concentration values are reported prior to Log transformation. sIgA concentration is reported in μg/mL, T is reported in pg/mL, and C is reported in ng/mL.

**Table 2:** Bivariate correlations for variables.

	1.	2.	3.	4.	5.	6.	7.	8.	9.	10.	11.	12.
1. Age	-----											
2. Facial attractiveness	−0.01	-----										
3. Health problems	−0.08	−0.28***	-----									
4. sIgA	−0.02	0.14†	−0.14†	-----								
5. Skin Fold	0.10	−0.28***	0.34***	−0.01	-----							
6. Sleep	−0.03	0.04	−0.09	−0.05	−0.05	-----						
7. Exercise	−0.01	0.20*	−0.24***	0.27***	−0.13†	0.05	-----					
8. Alcohol (drink/week)	−0.15†	0.08	0.09	0.01	−0.03	0.08	0.04	-----				
9. Smoking	0.01	−0.02	0.14†	−0.05	−0.01	0.01	−0.31***	0.02	-----			
10. Life Stress	0.01	−0.10	0.12	0.01	0.25***	−0.15†	0.08	0.03	−0.12	-----		
11. Testosterone (T)	−0.24***	0.10	−0.01	−0.02	−0.03	0.05	0.05	0.09	0.06	−0.03	-----	
12. Cortisol (C)	−0.21***	0.04	0.10	0.12	−0.08	−0.06	−0.16*	0.07	0.20*	0.05	0.33***	-----
13. T x C	−0.22***	0.02	0.11	0.11	−0.07	−0.05	−0.15†	0.09	0.20*	0.03	0.41***	0.98***

† = *p* < 0.01; * = *p* < 0.05; ** = *p* < 0.01; *** = *p* < 0.001.

**Figure 2:**
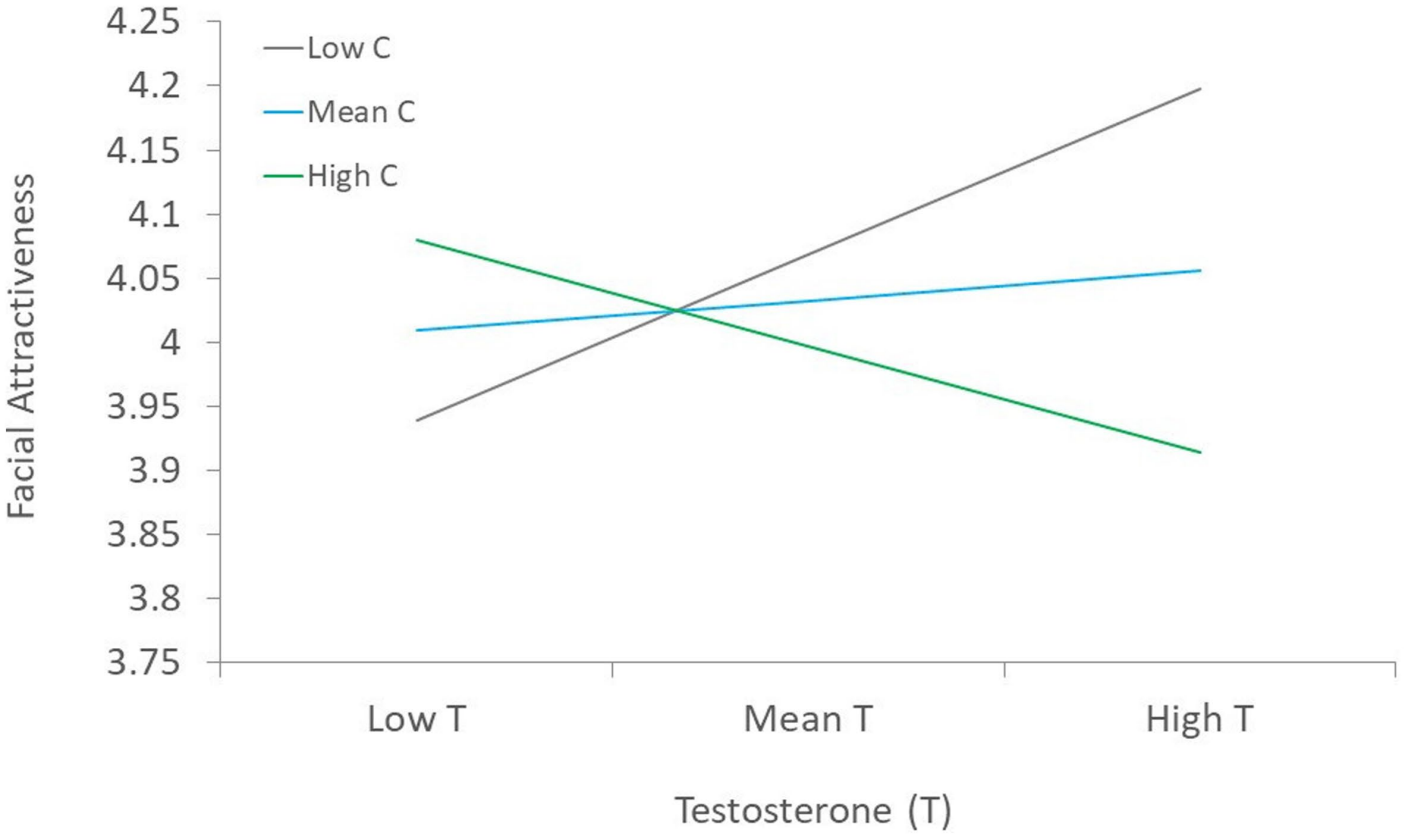
Visual depiction of the T x C interaction in a model predicting facial attractiveness.

Second, regression analysis was conducted with specific lifestyle indicators, skinfold, age, and hormones, entered as predictors of facial attractiveness simultaneously (Table 3) using Model 1 of the PROCESS macro for SPSS (Hayes, 2013). Results showed that average skinfold and symptoms of poor health^1^ were the only statistically significant predictors of facial attractiveness, such that poorer health and more body fat was linked to lower facial attractiveness. Although T, C, and the T x C interaction were not statistically significant predictors of facial attractiveness, these variables trended toward being statistically significant (e.g., *p*’s < 0.10).^2^ Visual examination of the interaction suggests a trend toward men with high T and low C being rated as more facially attractive (Figure 2).^3^

**Table 3:** Bootstrapped regression analyses (Model 1, PROCESS Macro) for lifestyle factors, skinfold, sIgA, testosterone and cortisol as predictors of men’s facial attractiveness (as rated by women).

	*B*	Std. Error	*t*	*p*	*R*^2^
DV: Facial attractiveness					0.18
Age	0.01	0.02	0.66	0.51	
Health Problems	−0.89	0.44	−0.20	0.04*	
Log sIgA	0.27	0.28	0.97	0.34	
Average skinfold (mm)	−0.03	0.01	−2.29	0.02*	
Sleep	−0.01	0.10	−0.01	0.99	
Exercise	0.42	0.29	1.45	0.15	
Alcohol (drinks/week)	0.02	0.02	1.26	0.21	
Smoking	0.11	0.47	0.23	0.82	
Life Stress	−0.16	0.25	0.63	0.53	
Testosterone (T)	0.21	0.52	0.40	0.69	
Cortisol (C)	−0.07	0.29	−0.23	0.82	
T x C	−1.18	0.70	−1.70	0.09†	

DV, Dependent Variable; B, Unstandardized regression coefficient. † = *p* < 0.10; * = *p* < 0.05.

Third, we considered the possibility that lifestyle factors might instead have an indirect effect on facial attractiveness, specifically via increased skinfold and associated health problems. To test this prediction, we used AMOS (version 29; Arbuckle, 2019) to create an observed variable path model, with facial attractiveness entered as the dependent variable, unhealthy lifestyle variables (family stress, smoking, drinking alcohol, exercise, and sleep) as the predictors, and abdominal skinfold and poor health symptoms as the sequential mediators. The chi-square test of significance (relative *χ*^2^ index values <3.00), Comparative Fit Index (CFI; values >0.90), and the root mean square error of approximation (RMSEA; values <0.08, Kline, 2016) were used to determine model fit. Indirect (mediation) effects were examined using 1,000 bootstrap samples and bias-corrected 95% confidence intervals. Results showed that, of the unhealthy lifestyle habits, life stress (*B* = 5.33, SE = 1.56, *β* = 0.25, *p* < 0.001) and exercise (*B* = −3.49, SE = 1.74, *β* = −0.16, *p* = 0.045) predicted abdominal skin fold, whereas smoking (*B* = −0.84, SE = 2.91, *β* = 0.02, *p* = 0.77), drinking alcohol (*B* = 0.04, *SE = 0.12*, *β* = −0.03, *p* = 0.74), and sleep (*B* = −0.06, SE = 0.66, *β* = −0.01, *p* = 0.93) did not. Skinfold, in turn, directly predicted the severity and frequency of poor health symptoms (*B* = 0.01, SE = 0.002, *β* = 0.34, *p* < 0.001). Poor health symptoms, in turn, predicted lower facial attractiveness (*B* = −1.05, SE = 0.41, *β* = −0.20, *p* = 0.01). Skinfold directly negatively predicted facial attractiveness (*B* = −0.03, SE = 0.01, *β* = −0.21, *p* = 0.007), and had an indirect effect through the mediator of poor health symptoms (*B* = −0.01, SE = 0.004, *p* = 0.015, 95% LLCI = −0.02, 95% ULCI = −0.003). Exercise (*B* = 0.15, SE = 0.09, *p* = 0.03, 95% LLCI = 0.02, 95% ULCI = 0.36) and life stress (*B* = −0.23, SE = 0.10, *p* = 0.02, 95% LLCI = −0.46, 95% ULCI = −0.06) also showed statistically significant indirect effects through the sequentially mediated pathway of skinfold and poor health symptoms upon facial attractiveness. The sequential mediation model fit the data well, relative *χ*^2^ index = 1.31 (*df* = 19, *p* = 0.16), RMSEA = 0.01 (95% CI = 0.00–0.08), CFI = 0.91 (Figure 1).

## 5 Discussion

Tests of the links between facial attractiveness and health have yielded mixed results, with some researchers suggesting that lifestyle factors common to both facial attractiveness and health might account for these links (Jones et al., 2021). Accordingly, the present study examined whether indicators of immunocompetence (self-reported poor health symptoms and sIgA), unhealthy lifestyle (smoking, alcohol consumption, poor sleep, lack of exercise, and family stress), age, along with skinfold (as an index of body fat) and hormones (testosterone and cortisol) predicted facial attractiveness. Initially, results showed a significant bivariate link between facial attractiveness and self-reported health symptoms, and this relationship remained statistically significant when including age, unhealthy lifestyle habits, along with skinfold, T, C, and the T x C interaction in the model. However, none of the lifestyle factors themselves predicted facial attractiveness, whereas skinfold did. Previous work has shown that abdominal skinfold is a strong predictor of both facial adiposity and overall facial attractiveness (Sierra-Johnson and Johnson, 2004; Windhager et al., 2011). Similar findings have been observed in women, where BMI predicts facial attractiveness (Han et al., 2016). These links are likely due to related changes to facial morphology that are associated with visceral body fat (Lee and Kim, 2014). Abdominal skinfold is strongly associated with diverse indices of poor health (see Lee and Kim, 2014 for review) and future all-cause mortality in white males (Loh et al., 2018). Skinfold is influenced by lifestyle factors, including nutrition and exercise (e.g., Kwak et al., 2010). We therefore considered whether lifestyle factors instead had an indirect effect upon facial attractiveness via abdominal skinfold and subsequent poor health. Results of a mediation analysis supported this for two of the lifestyle variables: Exercise and life stress. This finding suggests that exercise and life stress have an indirect effect on facial attractiveness via changes to body fat and related poor health symptoms. Some of the lifestyle factors examined here do not necessarily increase body fat. For example, although smoking has been linked to long-term weight gain (Carrasquilla et al., 2024), it may have more meaningful effects in young adulthood upon skin quality and specific health problems (e.g., lung disease). Given that only 6% of our sample smoked, we were likely unable to appropriately assess the potential indirect effects of smoking on facial attractiveness. Future research using a broader community-based sample could address this limitation.

There was also a modest positive correlation between female-rated facial attractiveness and men’s sIgA, but this effect was eliminated in the regression equation that included the control variables. This finding corresponds with that of Cai et al. (2019) who found that sIgA was broadly unrelated to female facial appearance. Unlike other sexually dimorphic features that have been linked to sIgA, such as male voice pitch (Arnocky et al., 2018; Hodges-Simeon et al., 2024) and female breast symmetry (Locke and Arnocky, 2021), this null finding could mean that links between health and facial attractiveness are weaker than with other attractive secondary sex characteristics, or perhaps are more strongly driven by lifestyle influences. Future work involving a broader range of immunological markers in relation to facial appearance is therefore encouraged.

Both T and C were also uncorrelated with men’s facial attractiveness. However, the regression equation controlling for other variables led to a modestly significant positive link between the T x C interaction and facial attractiveness. Specifically, men with higher T and lower C were rated as most attractive, but this effect did not reach the conventional benchmark for statistical significance. However, it is noteworthy that this finding does conform to that of Rantala et al. (2012), who found the same effect. The overall weak association between hormones and facial attractiveness diverges from a study of women which showed a link between high C and lower facial attractiveness (Rantala et al., 2013), but corresponds with others of male facial attractiveness showing no links with either hormone (Swaddle and Reierson, 2002; Neave et al., 2003; Penton-Voak and Chen, 2004; Kandrik et al., 2017; Kordsmeyer et al., 2019). It has long been assumed in evolutionary psychology that male facial attractiveness is an honest cue of an individual’s health and immunocompetence (see Jones et al., 2021 for discussion). Some work does support links between certain immune markers (e.g., high functioning natural killer cells) being associated with female perceptions of male facial attractiveness (Mengelkoch et al., 2022).

T and C did not correlate with symptoms of poor health or sIgA. These findings contrast with previous work on similar samples of young adult men from Northern Ontario that have shown positive links between single samples of sIgA and T (Arnocky et al., 2018). There is a need for more comprehensive assessments of hormonal markers in relation with health variables, perhaps by assessing ‘trait’ levels of these hormones across multiple timepoints and days (discussed by Davis and Arnocky, 2022).

### 5.1 Limitations

One limitation of this work is the use of a homogenous sample of young, primarily Caucasian, undergraduates. This segment of the population tends to be particularly healthy, relative to the broader population. This likely limited variability in lifestyle, which might partly account for the relatively weak predictive role of most lifestyle factors. For instance, College graduates eat healthier, smoke less, and exercise more (see Lawrence, 2017 for review). Similarly, the brief measurement of each lifestyle factor was also limiting. There exist longer form measures of diet quality (Warren-Findlow et al., 2017), drinking behavior (The Drinking Styles Questionnaire DSQ; Smith et al., 1995), tobacco use (e.g., Fagerström Test for Nicotine Dependence FTND; Heatherton et al., 1991), physical activity (Healey et al., 2020), and sleep quality (Yi et al., 2006). The measures used in the present study also asked participants to self-report their own health symptoms and behaviors, and it is important to consider the various sources of self-report bias that can influence this kind of data (e.g., recall bias; Van den Bergh and Walentynowicz, 2016). Moreover, young adult men were the target population in the current study. Therefore, we cannot say that the same results would apply to different age groups, such as older adult men (e.g., Ponholzer et al., 2005). Although this study was sufficiently powered, the sample size was also a limitation, with some researchers suggesting that stability of estimates requires a larger sample than what was achieved in this study (Schönbrodt and Perugini, 2013). Future work should therefore consider these links in larger and more heterogenous samples. Reliance on statistical non-significance may be limited when examining control variables in a regression model to determine whether health remains a meaningful predictor of facial attractiveness. Another limitation lies in the reliance upon assessments of overall facial attractiveness. Although ecologically valid, this measure does not identify specific phenotypic structures of the face that might be tied to either immunocompetence or the effects of an unhealthy lifestyle. Using explicitly facial-oriented variables (e.g., geometric morphometric analyses, GMM) could help to determine how specific facial features contribute proportionally to explained variance in attractiveness, health, and lifestyle. For example, GMM has recently been used to examine facial features in relation with men’s and women’s sociosexual orientation (Antar and Stephen, 2021).

Future research could examine the impact of both lifestyle factors and hormones during development (adolescence) on adult facial attractiveness. Indeed, some aspects of facial attractiveness are relatively changeable (e.g., such as those affected by current health), whereas other aspects are more stable, such as facial masculinity, which is heavily influenced by steroids and some aspects of immune function during early adolescence (see Foo et al., 2020). Measuring these relationships during adolescence and again during adulthood might help to clarify their unique contributions to facial attractiveness. Finally, it may be useful for future work to consider including a measure of lean muscle mass, such as flexed bicep circumference (see, e.g., Holzleitner et al., 2014) as it may be related both to lifestyle and hormonal factors and has been tied to women’s ratings of men’s attractiveness via modified facial stimuli (e.g., Lei et al., 2019).

## 6 Conclusion

Mixed findings characterize the research on the links between facial attractiveness, health, and immunocompetence in men (Jones et al., 2021), which has significant implications for evolutionary theories dealing with the purported ultimate explanations for attractive phenotypic traits (e.g., immunocompetence handicap hypothesis; Nowak et al., 2018). We add to this growing literature to help make sense of the equivocal findings by considering lifestyle and hormonal factors that might influence the links between facial attractiveness and immune function. As suggested by previous authors (Jones et al., 2021), we did find evidence that lifestyle habits (indirectly) and hormones appear to matter when studying the relations between facial attractiveness and immunocompetence. These insights help to advance our understanding of why certain phenotypic traits (e.g., facial characteristics) are regarded as attractive and what kind of information these attractive traits communicate to others, such as health status and lifestyle habits.

## Data availability statement

Publicly available datasets were analyzed in this study. This data can be found here: https://osf.io/adxj3.

## Ethics statement

The studies involving humans were approved by Nipissing University Research Ethics Board. The studies were conducted in accordance with the local legislation and institutional requirements. The participants provided their written informed consent to participate in this study.

## Author contributions

SA: Writing – review & editing, Writing – original draft, Supervision, Methodology, Funding acquisition, Formal analysis, Conceptualization. AD: Writing – review & editing, Writing – original draft, Formal analysis.

## Footnotes

^1^Given past research linking facial attractiveness to less respiratory illness, less antibiotic use, but not fewer stomach bugs in male (Boothroyd et al., 2013) and female participants (Gray and Boothroyd, 2012). We reran the model with only respiratory symptoms comprising the health problems variable. Results did not meaningfully change from those reported.

^2^Including saliva sample time of day as a covariate did not meaningfully alter the results reported herein.

^3^Excluding the participant case with an outlying (very high) T concentration did not meaningfully alter the results of the interaction results reported herein.
